# Development of lysozyme-combined antibacterial system to reduce sulfur dioxide and to stabilize Italian Riesling ice wine during aging process

**DOI:** 10.1002/fsn3.238

**Published:** 2015-04-28

**Authors:** Kai Chen, Shun-yu Han, Bo Zhang, Min Li, Wen-jun Sheng

**Affiliations:** Gansu Key Laboratory of Viticulture & Enology (Lanzhou), College of Food Science & Engineering, Gansu Agricultural UniversityLanzhou City, 730070, P. R. China

**Keywords:** Ice wine, LAB inhibition, lysozyme, protein stability, quadratic rotation-orthogonal composite design, response surface methodology, ridge analysisridge analysis

## Abstract

For the purpose of SO_2_ reduction and stabilizing ice wine, a new antibacterial technique was developed and verified in order to reduce the content of sulfur dioxide (SO_2_) and simultaneously maintain protein stability during ice wine aging process. Hazardous bacterial strain (lactic acid bacteria, LAB) and protein stability of Italian Riesling ice wine were evaluated in terms of different amounts of lysozyme, SO_2_, polyphenols, and wine pH by single-factor experiments. Subsequently, a quadratic rotation-orthogonal composite design with four variables was conducted to establish the multiple linear regression model that demonstrated the influence of different treatments on synthesis score between LAB inhibition and protein stability of ice wine. The results showed that, synthesis score can be influenced by lysozyme and SO_2_ concentrations on an extremely significant level (*P *< 0.01). Furthermore, the lysozyme-combined antibacterial system, which is specially designed for ice wine aging, was optimized step by step by response surface methodology and ridge analysis. As a result, the optimal proportion should be control in ice wine as follows: 179.31 mg L^−1^ lysozyme, 177.14 mg L^−1^ SO_2_, 0.60 g L^−1^ polyphenols, and 4.01 ice wine pH. Based on this system, the normalized synthesis score between LAB inhibition and protein stability can reach the highest point 0.920. Finally, by the experiments of verification and comparison, it was indicated that lysozyme-combined antibacterial system, which was a practical and prospective method to reduce SO_2_ concentration and effectively prevent contamination from hazardous LAB, can be used to stabilize ice wine during aging process.

## Introduction

Wine is a product made from a series of complex metabolism processes driven by different microbial species. Among these species, yeast and lactic acid bacteria (LAB) are the most important microorganisms initially present in vineyards and thrive throughout fermentation until packaging (Ribéreau-Gayon et al. 2006). In the absence of oxygen yeast converts the sugars of wine grapes into alcohol and carbon dioxide through the process of alcoholic fermentation (AF), and LAB are in charge of decarboxylation of *L*-malic acid to form *L*-lactic acid which provides the elegant and round taste during malolactic fermentation (MLF) (Tchelistcheff et al. 1971; Ribéreau-Gayon et al. 2006; Lopez et al. 2009). However, several LAB species, such as *L. brevis*, *L. plantarum*, and *P. damnosus* could elicit negative effects on wine quality (Fugelsang and Edwards 2006). Sometimes, LAB are considered hazardous because their metabolism is responsible for “lactic disease,” which is characterized by production of off-flavor compounds and biogenic amines (Ribéreau-Gayon et al. 2006).

Because of broad-spectrum antimicrobial and antioxidant activities, sulfur dioxide (SO_2_) is always regarded as a conventional and effective preservative which is necessary for LAB inhibition and MLF control in winemaking process (OIV 2009). However, this strategy may cause adverse effects, such as undesirable aroma of sulphurous gas and color loss in final wine products (Gerbaux et al. 1997). Meanwhile, this substance has already been applied in many foods processing for antiseptic purpose. Excess SO_2_ intake will cause cumulative toxicity on human organs, thereby resulting in headaches, nausea, and asthmatic reactions on sensitive individuals (Gao et al. 2002; Bartowsky et al. 2004). For this reason, The International Organization of Vine and Wine (OIV) issued SO_2_ limitation to avoid misuse for different winemaking scenarios (OIV 2009). Consequently, enologists are eager to develop some more natural and healthful SO_2_ alternatives to stabilize the wine quality.

Lysozyme, also known as muramidase, are glycoside hydrolases which is a type of enzyme (EC 3.2.1.17) that damages bacterial cell walls by catalyzing hydrolysis of 1,4-beta-linkages between N-acetylmuramic acid and N-acetyl-D-glucosamine residues in peptidoglycan (found in the cell walls of bacteria, especially gram-positive bacteria). (Charter and Lagarde 1999; Azzolini et al. 2010; Guzzo et al. 2011). Its application is a mature strategy for controlling lactic acid bacteria in food industry, especially for cheese processing. Moreover, for white wine making, Lysozyme is commonly known as an antimicrobial protein which can replace SO_2_ to delay MLF and control volatile acidity concentration. (Cejudo-Bastante et al. 2010). Some researchers also suggested that adding lysozyme is to prolong maceration and microoxygenation for improving color stabilization and polyphenolic extraction in red wine (Pilatte 2005). Until now, since no negative effects of lysozyme on sensory properties were found, maximum 500 mg L^−1^ lysozyme is allowed to use during wine aging process by OIV. (Ni and Zhong 2003; OIV 2009). However, it should be noticed that depending only on lysozyme to replace SO_2_ could cause oxidation and even browning in white wine because lysozyme does not possess the antioxidant activity (Pilatte et al. 2000; Matito et al. 2003; Pilatte 2005). Based on literatures and our research, compared with the use of enological tannins in white wine, (Sonni et al. 2009), the postfermentative addition of oligomeric proanthocyanidin (OPC), which is a set of bioflavonoid complexes that are extracted from grape seeds and perform as free radical scavengers in human body, can perform higher antioxidant and antibacterial activity and lower astringency (low hydrogen bonding with protein). (Fujii et al. 2007; Guzzo et al. 2011).

Hexi Corridor (Gansu Province, Northwest China) is a historic and major wine region in China. It is characterized by year-round dry and cold climate, fewer pest and disease problems, and Italian Riesling grapes, one of the primary white varieties in this region, are rich in fruity and elegant aromas (Song et al. 2009; Cui et al. 2012). However, the optimum quality of ice wine is unlikely achieved when high SO_2_ concentration (>500 mg L^−1^) is added during aging progress (Gao and Liu 2008; Li et al. 2012). An appropriate vinification technique should be developed to improve aging quality of Italian Riesling ice wine. According to the previous studies, postfermentative addition of lysozyme combined with OPC can be taken into account as a promising technique to modify inherent flaws.

The development of lysozyme-combined antibacterial system aims to help winemakers to reduce SO_2_ addition during aging process and to use postfermentative lysozyme in a more efficient way.

## Materials and Methods

### Wine samples

Italian Riesling ice wine was supplied by Qilian winery (Gansu, China). The grapes were harvested in 2012 vintage (November 3^rd^–6^th^) at full maturity (soluble solids 35–36 °Brix, total acidity 8–9 g L^−1^, and pH 3.5) and in good sanitary conditions. The wine was made with a traditional ice-wine-processing protocol: grapes were pressed in a pneumatic press and 80 mg L^−1^ sulfur dioxide and 35 mg L^−1^ pectinolytic enzyme (Ex-pression, Enartis, Italy) were added afterward. The grape juice was then settled at 10°C for 12 h. The alcoholic fermentation was induced by inoculating *Saccharomyces cerevisiae* (LVCB, 30 g·h L^−1^, 25°C, Enartis, Italia) and occurred at controlled temperature (10–12°C). At the end of alcoholic fermentation, 50 L of wine samples were immediately transported to the laboratory and stored at 0°C for further use. All wine samples were filter-sterilized through 0.65 *μ*m pore size of polycarbonate membrane of Tong-Wang TWML04 membrane filter (Hefei, China) before experiments. Experimental bottles were pregassed with food-grade nitrogen gas before filling to prevent microbial contamination and oxygen entrance.

### Chemicals and standards

Lysozyme chloride was supplied by Asahi Kasei Corporation (Tokyo, Japan), while the LAB (*Lactobacillus plantarum* VT12 and *Oenococcus oeni* VP41) used in this work were, respectively, purchased from Martin Vialatte Oenologie and Lallemand Oenology, France. Potassium metabisulfite and potassium hydrogen tartrate were purchased by Kemiou Chemical Reagent Co. (Tianjin, China). In order to simulate different winemaking scenarios, Oligomeric Proanthocyanidin (OPC), which was obtained from JF-Natural industry (Tianjin, China), was used to adjust the concentration of total polyphenols in ice wine. Folin–Ciocalteu reagent was supplied by Sigma-Aldrich chemical Co. (St. Louis, MO). Deionized water (<18 MW resistance) was obtained from a Milli-Q Element water purification system (Millipore, Bedford, MA).

### Enological parameters analysis

The principle physicochemical parameters in ice wine were detected at just the end of alcoholic fermentation, according to the China national quality standard of vine and wine (GB/T 15038 – 2006). These parameters included alcohol degree, residual sugar, total acids, volatile acids, total sulfur dioxide, *L*-malic acid, color density, wine pH, total phenols and tannin concentrations. As a result, low volatile acidity (0.36 mg L^−1^) and alcohol degree (11.2–13.8% v/v) combined with the residual sugars (188.2 g L^−1^) denoted the proper fermentative condition. (Li et al. 2006). In addition, total acidity (6.8 g L^−1^ as tartaric acid), wine pH 4.27, total polyphenols 0.41 g L^−1^, and total sulfur dioxide 49.2 mg L^−1^ (<60.0 mg L^−1^) were detected as basic indexes after AF.

### LAB culture and colonies counting

Spontaneous LAB and commercial LAB (*L. plantarum* VT12 and *O. oeni* VP41 mixed by 1:1 inoculation ratio) were enumerated under 37°C in liquid MRS medium which included 50 mg L^−1^ pimaricin and the initial pH was adjusted to 5.0 with a concentrated (6 molL^−1^) hydrochloric acid solution (Gao et al. 2002). About 1 mL cell suspension of LAB was used to contaminate 500 mL ice wine, and then LAB were cultured at 37 ± 2°C until the final inoculation size over 10^7^ CFU mL^−1^. Subsequently, the contaminated ice wine samples were carried out in resistance test against lysozyme and single-factor experiments.

LAB colonies quick-counting method was carried out to modify plate colonies counting according to De-qing Zhou (De-qing Zhou 2006). Of 10-mL tubes containing 9 mL modified semisolid MRS medium at pH 5.0 with the addition of 50 mg L^−1^ pimaricin and 0.3–0.4% agar were heated at 50 °C to melt. About 1 mL of contaminated ice wine sample was diluted by 9 mL semisolid MRS medium until 10^−7^. Each experimental group was repeated three times. Finally, these tubes were placed vertically on the tube racks and incubated at 37°C about 24 h. The spherical and lenticular LAB colonies were counted in valid tubes (30–300 CFU included). The total LAB colonies in ice wine samples were calculated with the formula 1 stated below:

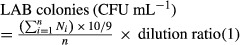


In this formula, *n* stands for the number of valid tubes for calculating, and N stands for the CFU in every tube.

### Heat test for protein stability

The level of protein stability was determined by heat test (Pocock et al. 2003) in order to show the level of protein stability with heat treatments. Before test, the absorbance A_0_ of each sample was detected at 540 nm and then 10 mL treatment was sealed in test tubes with screw caps. Subsequently, test tubes were heated in water baths at 80°C for 6 h and held at 4°C for 16 h afterward. Absorbance *A*_1_ was detected at room temperature. The change in absorbance, ΔOD_540_ (*A*_1_ − *A*_0_) was measured by Thermo GENESYS™ 10S UV-Vis spectrophotometer (Thermo Scientific Inc., San Joes, CA). Wines were considered to be stable if the difference in absorbance did not exceed 0.2 units. All the treatments were made in triplicate.

### Experimental design

First of all, four variables (lysozyme concentration, SO_2_ concentration, polyphenol concentration, and ice wine pH) which can affect the LAB inhibition and protein stability with common influences were studied, respectively, by single-factor experiments. Then, the quadratic rotation-orthogonal composite design with four factors at five levels was used to establish the multiple regression model that expressed the influence of different treatments on the synthesis score between LAB inhibition and protein stability.

#### Single-factor experiments of effect on LAB inhibition and protein stability

(1) To understand the influence of lysozyme, 0 mg L^−1^, 50 mg L^−1^, 100 mg L^−1^, 150 mg L^−1^, 200 mg L^−1^, and 250 mg L^−1^ were added into 500 mL ice wine samples, respectively; (2) To understand the influence of SO_2_, its concentration was adjusted to 50 − 250 mg L^−1^ by potassium metabisulfite addition; (3) To understand the influence of polyphenols, 500 mL ice wine samples were supplemented with OPC to reach total polyphenols content at 0.4 g L^−1^, 0.5 g L^−1^, 0.6 g L^−1^, 0.7 g L^−1^, and 0.8 g L^−1^; (4) 3.0 − 5.0 wine pH was studied with adjustment of potassium hydrogen tartrate. All samples were stored 5 days at 25°C for LAB development and then conducted for LAB inhibition and protein stability. Each sample was made in triplicate.

#### Quadratic rotation-orthogonal composite design

The quadratic rotation-orthogonal composite design (Table1) is a comprehensive and accurate statistical tool for multiple linear regression analysis (Zhan et al. 2013). Not only does it provide specific information from all treatments but also reveals inherent changes of interactions with the least experimental trials (Yu and Wang 2007; Jiao et al. 2008). Based on the results from single-factor experiments, a quadratic rotation-orthogonal composite design with four factors at five levels was conducted (Table2) to evaluate synthesis score for 36 experimental groups. In terms of precision, 12 replicate runs at central points of the design were performed to estimate pure errors (Jiao et al. 2008).

**Table 1:** Quadratic orthogonal-rotation composite design with the corresponding coded factors for variable levels

Coded level	*x*_1_ lysozyme/(mg L^−1^)	*x*_2_ SO_2_/(mg L^−1^)	*x*_3_ wine polyphenols/(g L^−1^)	*x*_4_ ice wine pH
−2	50	50	0.4	3
−1	100	100	0.5	3.5
0	150	150	0.6	4
1	200	200	0.7	4.5
2	250	250	0.8	5
Δ *j*	50	50	0.1	0.5

**Table 2:** Corresponding experimental results of quadratic rotation-orthogonal composite design

Test number	Single-factor code				Response value				
	Lysozyme *x*_1_	SO_2_ *x*_2_	Polyphenols *x*_3_	Ice wine pH *x*_4_	log10 CFU mL^−1^ *y*_1_:	ΔOD_540_ *y*_2_:	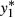 1	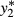 2	Synthesis Score^3^
1	−1	−1	−1	−1	3.29	0.158	0.211	0.649	0.386
2	1	−1	−1	−1	1.64	0.232	0.607	0.169	0.432
3	−1	1	−1	−1	2.35	0.149	0.436	0.708	0.545
4	1	1	−1	−1	0	0.236	1.000	0.143	0.657
5	−1	−1	1	−1	3.42	0.232	0.180	0.169	0.175
6	1	−1	1	−1	2.67	0.217	0.360	0.266	0.322
7	−1	1	1	−1	2.64	0.146	0.367	0.727	0.511
8	1	1	1	−1	0	0.219	1.000	0.253	0.701
9	−1	−1	−1	1	4.17	0.198	0.000	0.390	0.156
10	1	−1	−1	1	2.68	0.231	0.357	0.175	0.285
11	−1	1	−1	1	2.64	0.174	0.367	0.545	0.438
12	1	1	−1	1	0	0.212	1.000	0.299	0.719
13	−1	−1	1	1	2.19	0.172	0.475	0.558	0.508
14	1	−1	1	1	2.11	0.179	0.494	0.513	0.502
15	−1	1	1	1	2.06	0.232	0.506	0.169	0.371
16	1	1	1	1	0	0.188	1.000	0.455	0.782
17	−2	0	0	0	2.74	0.168	0.343	0.584	0.440
18	2	0	0	0	0	0.219	1.000	0.253	0.701
19	0	−2	0	0	3.18	0.236	0.237	0.143	0.200
20	0	2	0	0	0	0.258	1.000	0.000	0.600
21	0	0	−2	0	1.47	0.191	0.647	0.435	0.563
22	0	0	2	0	1.62	0.195	0.612	0.409	0.531
23	0	0	0	−2	1.26	0.206	0.698	0.338	0.554
24	0	0	0	2	1.83	0.192	0.561	0.429	0.508
25	0	0	0	0	0.76	0.161	0.818	0.630	0.743
26	0	0	0	0	0.15	0.131	0.964	0.825	0.908
27	0	0	0	0	0.68	0.147	0.837	0.721	0.790
28	0	0	0	0	0.27	0.152	0.935	0.688	0.836
29	0	0	0	0	0	0.136	1.000	0.792	0.917
30	0	0	0	0	0.39	0.145	0.906	0.734	0.837
31	0	0	0	0	0	0.142	1.000	0.753	0.901
32	0	0	0	0	0.89	0.142	0.787	0.753	0.773
33	0	0	0	0	0.67	0.104	0.839	1.000	0.904
34	0	0	0	0	0.35	0.123	0.916	0.877	0.900
35	0	0	0	0	0	0.115	1.000	0.929	0.971
36	0	0	0	0	0.72	0.113	0.827	0.942	0.873

1 The data of 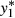 column were normalized from *y*_1_ log10 CFU mL^−1^ (LAB inhibition) according to formula 2.

2 The data of 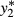 column were normalized from y_2_ ΔOD_540_ (protein stability) according to formula 2.

3 Synthesis score was the normalized results that were summed according to formula 3 by weighting LAB inhibition as 60% and protein stability as 40%, respectively.

#### Linear transformation of raw data with min–max normalization

Min–max normalization is a statistical method, which is used for linear transformation of the original data and makes the result value mapped into the range of 0−1. In this way, the restriction of raw data units can be removed, and the raw data are converted into pure dimensionless value, which facilitates the indicators of different units or magnitudes that can be compared and weighted (Zhang et al. 2011). Min-max normalization was carried out to synthetically evaluate the lysozyme-combined antibacterial system and includes two various indexes both ΔOD_540_ (for protein stability) and log10 CFU mL^−1^ (for LAB inhibition). Conversion functions are as follows.

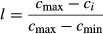
2

In formula 2, *c*_*i*_ was index value, *c*_min_ was minimum index value, and *c*_max_ was maximum index value.

Synthesis score of the antibacterial system was obtained by weighting log10 CFU mL^−1^ as *a* =* *60% (primary index) and ΔOD_540_ as *b* =* *40% (secondary index). In formula 3, *l*_*v*_ was the dimension of LAB inhibition and *l*_*e*_ was the dimension of protein stability. Letters *“a”* and *“b”* stand for the two weights.

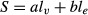
3

### Statistical analysis

The multiple regression model and multiple-way analysis of variance (ANOVA) were analyzed by SAS 9.0 software (SAS Institute Inc., Cary, NC). Tukey's test was carried out to express group differences at significance level *P *<* *0.05. Subsequently, SAS 9.0 was used to generate response surfaces and contour plots for the relationships between two variables. In order to modify the multiple regression model (formula 4), ridge analysis was applied to determine the optimal combination for lysozyme-combination antibacterial system.

## Results and Discussion

### LAB resistance tests

**Figure 1:**
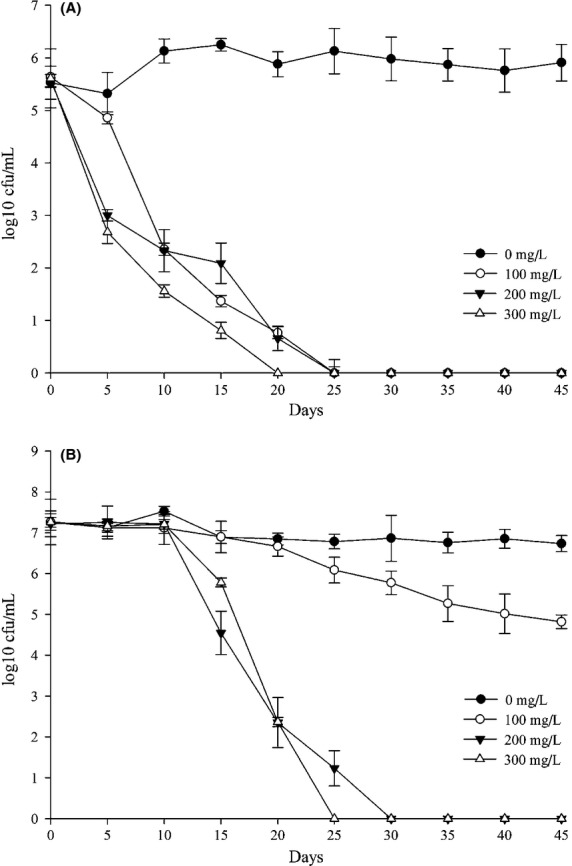
Inhibition of lysozyme on spontaneous LAB (A) and commercial LAB (B) (L. plantarum VT12 and *O. oeni* VP41 in 1:1 inoculation ratio). LAB inhibition was measured during 45 days in contaminated ice wine with the lysozyme addition of 0 (full circle), 100 (empty circle), 200 (full triangle), and 300 (empty triangle) mg L^-1^. Bars showed standard deviations.

Lysozyme-induced inhibition of spontaneous LAB in ice wine was illustrated in Fig.1A. Spontaneous LAB can be inhibited by 100–200 mg L^−1^ lysozyme addition in approximately 25 days. However, spontaneous LAB activity was completely eliminated in 20 days when lysozyme concentration was increased to 300 mg L^−1^.

In contrast, the mixed strain (inoculation ratio 1:1) of *L. plantarum* VT12 and *O. oeni* VP41, which are the typical and numerous LAB strains for ice wine making, was completely eliminated by 200–300 mg L^−1^ lysozyme in 25–30 days (Fig.1B). Since *L. plantarum* is a type of spoilage LAB which possess high resistance against lysozyme (Azzolini et al. 2010), the inhibitory effect was not significant when 100 mg L^−1^ lysozyme acted on the mixed strain, about 10^5^ CFU mL^−1^ LAB colonies still can be observed after 45 days. However, *O. oeni, which* dominates in the initial fermentation stage and gradually disappears at the end of alcoholic fermentation, showed the converse growth trend from *L. plantarum* to against lysozyme; about 100 mg L^−1^ lysozyme can effectively inhibit the activity of *O. oeni* (Lopez et al. 2009; Azzolini et al. 2010). As a result, *L. plantarum* VT12 and *O. oeni* VP41, two types of commercial LAB, were selected as target microbes and used to conduct a series of experiments during ice wine aging.

### Single-factor experiments on LAB inhibition and protein stability

**Figure 2:**
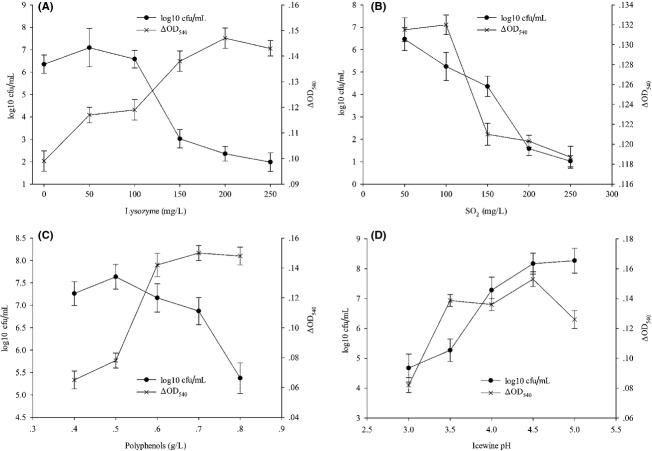
Influence of different concentration of lysozyme (A), SO2 (B), polyphenols (C), and wine pH (D) on LAB inhibition and protein stability. LAB inhibition index log10 CFU mL^−1^ (full circle) and protein stability index ΔOD540 (cross) were measured at pH 4.27 in the ice wine contaminated with L. plantarum VT12 and O. oeni VP41 (1:1 inoculation ratio). Bars showed standard deviations.

Base on Fig.2A, LAB activity decreased as lysozyme concentration increased. LAB cannot be inhibited by less than 100 mg L^−1^ lysozyme. However, 150 mg L^−1^ lysozyme significantly affected LAB activity (log10 CFU mL^−1^), which reduced by 57.3% as comparing with 100 mg L^−1^ lysozyme. Therefore, LAB growth can be effectively inhibited by 150 mg L^−1^–200 mg L^−1^ lysozyme addition. The protein stability of ice wine was enhanced as the amount of lysozyme was increasing. For instance, ΔOD_540_ kept relatively stable at the level of 50–100 mg L^−1^ lysozyme and then increased dramatically when the addition was increasing from 100 mg L^−1^ to 200 mg L^−1^ until the highest point at 0.147 with 200 mg L^−1^ lysozyme. However, ΔOD_540_ decreased slightly at the range of 200–250 mg L^−1^ lysozyme. Figure2A indicates that 100–200 mg L^−1^ lysozyme can significantly affect the protein stability of Italian Riesling ice wine.

Same methods and LAB strains were used to monitor the changes of LAB activity under different SO_2_ concentrations. Figure2B illustrated that LAB activity decreased as SO_2_ concentration increased, 33.9% LAB activity was inhibited by 50–150 mg L^−1^ SO_2_ addition. LAB inhibition at 200 mg L^−1^ SO_2_ content was about twice higher than that at 150 mg L^−1^ SO_2_. This result indicated that 150–250 mg L^−1^ SO_2_ can significantly affect LAB growth in Italian Riesling ice wine. With the same gradient of SO_2_, heat test was conducted to detect the protein stability. Figure2B showed that ΔOD_540_ decreased as SO_2_ was increased from 0 mg L^−1^ to 150 mg L^−1^. A considerable decrease was observed when SO_2_ concentration was 100–150 mg L^−1^, which could be a hazardous level to the protein stability. But above 150 mg L^−1^ SO_2_ seems moderate for the change in the protein stability.

Although the addition of lysozyme would be ineffective to control LAB because of high affinity binding between polyphenols and proteins (Bartowsky et al. 2004; Rawel and Rohn 2010). An appropriate concentration of polyphenols in white wine can not only protect the wine from oxidation but also increase the antibacterial activity for LAB contamination (Waters et al. 2005). Moreover, the addition of oenological tannins replacing SO_2_ showed no negative effects on the fermentative process, providing even a better sensory perception when compared with the wine having only SO_2_ addition (Rolle et al. 2008; Segade et al. 2008). In this work, OPC, which possesses similar prosperities with oenological tannins but with lighter color, was used as a complement for adjustment of total polyphenols. The effect of total polyphenol on LAB inhibition and protein stability was studied according to Fig.2C. LAB activity was inhibited as polyphenol content increased from 0.5 g L^−1^ to 0.8 g L^−1^. Because of the antimicrobial properties of phenolic compounds, 38.7% LAB activity was inhibited by 0.7 g L^−1^ polyphenols content. Overall, over 0.6 g L^−1^ polyphenols can effectively affect LAB activity after alcoholic fermentation. In terms of the change in protein stability, ΔOD_540_ increased as total polyphenols increased from 0.4 g L^−1^ to 0.8 g L^−1^. Particularly, ΔOD_540_ double increased when 0.6 g L^−1^ total polyphenols was adjusted. However, the tendency was becoming to be gradually moderate at 0.6 g L^−1^–0.8 g L^−1^ polyphenol level. As a result, 0.6 g L^−1^ total polyphenols was taken into account as an effective concentration to maintain the protein stability of ice wine.

As far as we know, the optimum pH for LAB growth is 5.5–6.0 in liquid medium (Bartowsky 2009). Indeed, LAB activity was positively correlated with pH increasing in Fig.2D. LAB colonies significantly increased from 10^5^ CFU mL^−1^ to 10^7^ CFU mL^−1^ at pH 3.5–4.0, about 40.4% log10 CFU mL^−1^ increased, which could be extremely hazardous for ice wine aging with low SO_2_ addition. In terms of the protein stability, ΔOD_540_ increased as pH was increasing from 3.0 to 3.5, with a slight decrease from 0.140 to 0.136 and subsequently increased to the highest point 0.151 at pH 4.5. In the end, ΔOD_540_ decreased to 0.126 at pH 5.0. Considering about wine practical pH value, 3.5–4.0 was taken into account as the suitable range for the antibacterial system building.

### Multiple regression model and ANOVA analysis

Quadratic rotation-orthogonal composited design (Table2) was carried out with four variables, namely, lysozyme contents (*x*_1_), SO_2_ contents (*x*_2_), polyphenol contents (*x*_3_), and pH-value (*x*_4_). LAB inhibition (log10 CFU mL^−1^) was fixed as *y*_1_ and ΔOD_540_, the index of protein stability was fixed as *y*_2_. 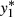 and 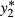 represented the dimensions that were converted from the original data by min-max normalization. Subsequently, a multiple regression model of synthesis score was established to analyze the stabilization level of Italian Riesling ice wine. The response surface methodology (RSM) was used to evaluate the optimal range of different factors. In order to take further optimization, data collinearity was eliminated by a ridge analysis.

SAS 9.0 was used to carry out experimental data processing for multiple linear regressions. A quadratic polynomial regression equation (formula 4) with key controlling factors was established.

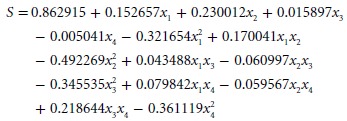
4

ANOVA results are shown in Table3. The lack of fit value, *F*_Lf_ = 2.04 < *F*_0.05_ (10, 11) = 2.8655, was not significant at *α *= 0.05,while, total regression *F*_Tr_ = 18.11 > *F*_0.05_ (14, 21) = 2.209, was significant at *α *= 0.05. Thus, the regression model was logically fitted into experimental data. It demonstrated that the multiple regression model not only reflected the practical treatments accurately but also can be used to evaluate the influences of lysozyme-combined antibacterial system on both LAB inhibition and protein stability during ice wine aging.

**Table 3:** ANOVA analysis of multiple regression model and coefficient significance test

Source of variation		Degree of freedom	Quadratic sum	Mean square	*F*-value	*P*-value	Significance
Linear	*x*_1_	1	0.1398	0.1398	20.3740	0.0002	**
	*x*_2_	1	0.3174	0.3174	46.2537	<0.0001	**
	*x*_3_	1	0.0015	0.0015	0.2210	0.6432	
	*x*_4_	1	0.0002	0.0002	0.0222	0.8829	
Interaction items	*x*_1_*x*_2_	1	0.0289	0.0289	4.2131	0.0528	
	*x*_1_*x*_3_	1	0.0019	0.0019	0.2756	0.6051	
	*x*_1_*x*_4_	1	0.0064	0.0064	0.9289	0.3461	
	*x*_2_*x*_3_	1	0.0037	0.0037	0.5421	0.4697	
	*x*_2_*x*_4_	1	0.0035	0.0035	0.5170	0.4800	
	*x*_3_*x*_4_	1	0.0478	0.0478	6.9658	0.0153	*
Quadratic items	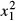	1	0.2069	0.2069	30.1510	<0.0001	**
	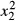	1	0.4847	0.4847	70.6204	<0.0001	**
	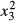	1	0.2388	0.2388	34.7943	<0.0001	**
	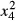	1	0.2608	0.2608	38.0037	<0.0001	**
Error		21	0.1442	0.0069			
Regression	Linear	4	0.4584	0.2432	16.69	<0.0001	**
	Quadratic item	4	1.1901	0.6314	43.33	<0.0001	**
	Interaction item	6	0.0922	0.0489	2.24	0.0794	
	Total regression	14	1.7407	0.9235	18.11	<0.0001	**
Residual errors	Lack of fit	10	0.0937	0.0094	2.04	0.1282	
	Random error	11	0.0504	0.0046			
	Total residual error	21	0.1442	0.0069			
Sum		35	1.8848				

**Extremely significant (*P *<* *0.01), * significant (*P *<* *0.05).

On the other hand, synthesis score was significantly affected (*P *< 0.01) by lysozyme and SO_2_ addition, whereas the influence of polyphenols and ice wine pH was not significant. Furthermore, only the interaction between polyphenols and pH was significant (*P *< 0.05). According to the numerical magnitude of standardized regression coefficient, the sequence of influence of the four factors on synthesis score was SO_2_ > lysozyme > polyphenols > pH.

### Response Surface Methodology of lysozyme-combination antibacterial system

The influence of interactions on synthesis score was analyzed in the method of RSM which was established based on the multiple regression model (formula 4).

**Figure 3:**
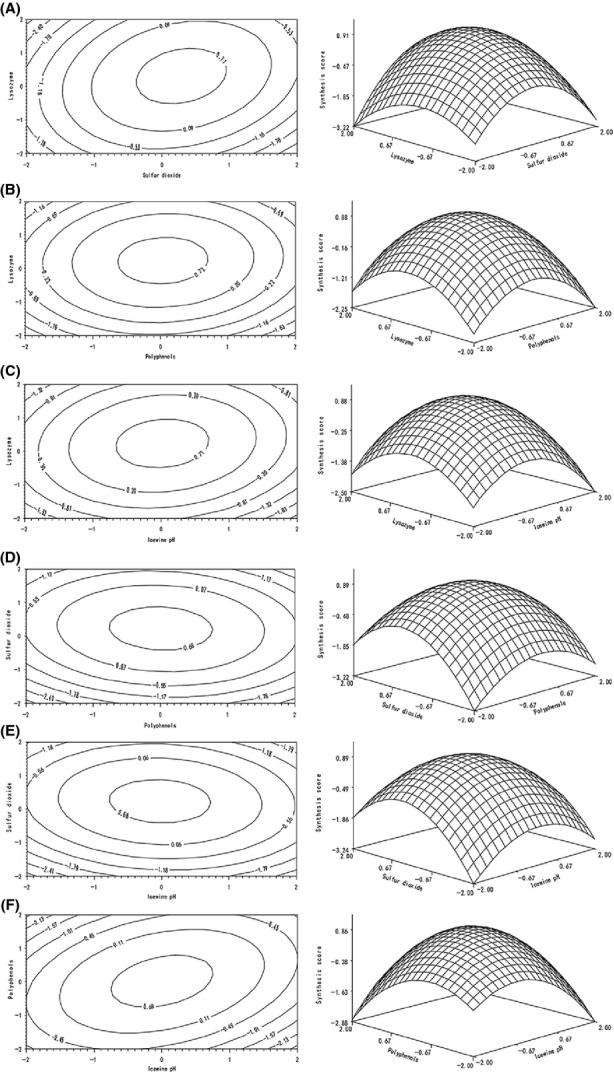
Response surfaces and contour plots (A–F) showed the influence of interaction of lysozyme (*x*_1_), SO2 (*x*_2_), polyphenols (*x*_3_), and wine pH (*x*_4_) on synthesis score. (A) Lysozyme and Sulfur dioxide (fixed factors: *x*_3_ = 0, *x*_4_ = 0); (B) Lysozyme and Polyphenols (fixed factors: *x*_2_ = 0, *x*_4_ = 0); (C) Lysozyme and wine pH (fixed factors: *x*_2_ = 0, *x*_3_ = 0); (D) Sulfur dioxide and polyphenols (fixed factors: *x*_1_ = 0, *x*_4_ = 0); (E) Sulfur dioxide and wine pH (fixed factors: *x*_1_ = 0, *x*_3_ = 0); (F) Polyphenols and wine pH (fixed factors: *x*_1_ = 0, *x*_2_ = 0).

With the increasing lysozyme and SO_2_ contents, respectively, the synthesis score initially increased and then decreased according to Fig.3A The highest point of synthesis score was over 0.91 with 150–200 mg L^−1^ lysozyme and 160–200 mg L^−1^ SO_2_. Excess SO_2_ addition (above 200 mg L^−1^) resulted in pH reduction and inhibited the activity of lysozyme (Ribéreau-Gayon et al. 2006; Azzolini et al. 2010; Guzzo et al. 2011). Meanwhile, low SO_2_ contents (less than 160 mg L^−1^) weakened the LAB inhibition. Based on Fig.3B, synthesis score initially increased and then decreased with increasing lysozyme and polyphenols contents, respectively, and the highest point was over 0.88 and in terms of polyphenols was 0.6–0.8 g L^−1^ and lysozyme was 125–250 mg L^−1^. It was due to the inhibition of lysozyme activity and even deactivation by protein convergence of excess polyphenols. On the contrary, because of low polyphenols (<0.6 g L^−1^), LAB reproduced dramatically, meanwhile, the antioxidant activity and stability of ice wine were affected simultaneously (Tirelli and De Noni 2007). Similar change in contour plot is shown in Fig.3C, the synthesis score reached at the highest point over 0.88 with 4–4.3 pH and 125–180 mg L^−1^ lysozyme contents. According to the ANOVA on Table3, the interactions between SO_2_, pH, and polyphenols are not significant to affect the synthesis score. The changes in synthesis score on Fig.3D–E were similar to Fig.3C, the highest points were all over 0.89 with 0.6–0.8 g L^−1^ total polyphenols, 3.5–4 pH and 125–250 mg L^−1^ SO_2_ contents.

According to Fig.3F, with fixed 0.6 g L^−1^ polyphenols, synthesis score peaked gradually and then decreased as pH increases at the range of 3.5–4.5. One hand, high wine pH (above 3.8) induced more bonded SO_2_ and attenuated the inhibitory effects on LAB, while lysozyme activity was inhibited by low pH (below 3.5). On the other hand, high concentration of polyphenols strongly bonded with lysozyme and induced protein haze in white wine (Bartowsky et al. 2004; Hua 2007; Guzzo et al. 2011). As a result, the synthesis score was influenced significantly by the interaction of polyphenols (0.6 g L^−1^) and pH (3.5–4.5) in ice wine.

Response surface methodology verified the conclusion of ANOVA in Table3; however, because of multicollinearity, which was mainly an interactive influence in arguments, the negative value could affect the precision in regression coefficient (Liyana-Pathirana and Shahidi 2005; Liu et al. 2014). In order to find out the optimal solution, the ridge analysis was carried out to further modify the multiple regression model.

### Ridge analysis

A ridge analysis was initially suggested by Hoerl (2007) in the context of fitted second-order response surface models where the factors were not restricted. Some theoretical foundation for the method was later given by Draper (). The basic method defines a series of paths outward from the origin (*x*_1_, *x*_2_, …, *x*_*q*_) = (0, 0, …, 0) of the factor space (Hoerl 1985; Draper and Pukelsheim 2000).

The results of ridge analysis were shown in Table4. The coded radius of the largest estimated response was 0.4, and the optimal solution of synthesis score was 0.920. In this case, an optimal lysozyme-combined antibacterial system can be realized by the following parameters: 179.31 mg L^−1^ lysozyme, 177.14 mg L^−1^ SO_2_, 0.60 g L^−1^ polyphenols, and pH 4.01. Thus, this system could reduce SO_2_ theoretically in ice wine and simultaneously maintain protein stability and LAB inhibition during aging process. For the purpose of practical application, the system was examined by the following validation test and comparative test during 6 months storage.

**Table 4:** Ridge analysis results for the synthesis score with different treatments

Coded radius	Estimated response	Standard Error	*x*_1_	*x*_2_	*x*_3_	*x*_4_
0.0	0.862750	0.023919	150.000000	150.000000	0.600000	4.000000
0.1	0.886845	0.023926	156.139142	157.877461	0.600977	3.998655
0.2	0.904142	0.024034	163.263213	164.942893	0.601775	3.999008
0.3	0.914986	0.024493	171.060733	171.322631	0.602647	4.001995
0.4	0.919619	0.025690	179.311928	177.137347	0.603835	4.008356
0.5	0.918214	0.028048	187.868228	182.476834	0.605563	4.018750
0.6	0.910911	0.031891	196.624121	187.398060	0.608037	4.033808
0.7	0.897826	0.037367	205.495543	191.930798	0.611444	4.054139
0.8	0.879067	0.044477	214.405984	196.085029	0.615950	4.080275
0.9	0.854744	0.053145	223.279345	199.858903	0.621676	4.112589
1.0	0.824964	0.063284	232.038998	203.247072	0.628687	4.151201

### Validation test for the optimal parameters

The obtained optimal parameters were subjected to validation tests. All the experimental data were the average value of the three replicated tests. According to the practical validation, LAB activity was completely inhibited and ΔOD_540_ was 0.121. The average error of the three replicated tests was under 0.05%. The standard deviations of two variables were 2.43% and 1.58%, respectively, which were close to theoretical values. Thus, the multiple regression model of the proposed system was correct and this modified lysozyme application for SO_2_ reduction, can be used to stabilize ice wine during aging process.

### Effect of lysozyme-combined antibacterial system on major physical–chemical compositions

In terms of major physical–chemical compositions, LAB inhibition and protein stability, the control group and the optimal group (lysozyme-combined antibacterial system) were monitored during 6 months aging in bottles. The results were shown on Table5.

**Table 5:** Comparative tests on LAB colonies, protein stability, and major physical–chemical compositions in Italian Riesling ice wine during 6 months of aging processing

Experimental parameters	Control group			Optimal group		
	2 months	4 months	6 months	2 months	4 months	6 months
Turbidity (OD_420_)	0.037 ± 0.001^a^	0.042 ± 0.001^b^	0.046 ± 0.001^c^	0.056 ± 0.001^a^	0.057 ± 0.001^a^	0.057 ± 0.002^a^
Total sugar (g L^−1^)	183.36 ± 3.85^b^	177.06 ± 1.63^b^	171.47 ± 1.81^a^	187.7 ± 0.42^a^	186.4 ± 0.70^a^	186.2 ± 2.36^a^
pH	4.3 ± 0.01^a^	4.5 ± 0.04^b^	4.6 ± 0.01^c^	4.01 ± 0.01^a^	4.02 ± 0.01^b^	4.02 ± 0.01^ab^
Total acidity (g L^−1^)	8.44 ± 0.21^a^	8.67 ± 0.08^ab^	9.13 ± 0.41^b^	6.32 ± 0.06^a^	6.36 ± 0.16^a^	6.25 ± 0.12^a^
Volatile acidity (g L^−1^)	1.41 ± 0.07^a^	1.73 ± 0.06^b^	1.98 ± 0.10^c^	0.38 ± 0.04^a^	0.37 ± 0.01^a^	0.37 ± 0.01^a^
Alcoholic degree (% vol)	12.6 ± 0.6^b^	12.2 ± 0.5^a^	11.3 ± 0.3^a^	12.5 ± 0.1^a^	12.6 ± 0.05^a^	12.6 ± 0.01^a^
SO_2_ (mg L^−1^)	47.73 ± 1.59^c^	42.33 ± 2.18^b^	30.1 ± 1.80^a^	178.25 ± 2.06^a^	175.41 ± 4.68^a^	175.13 ± 2.42^a^
Color density	1.27 ± 0.001^a^	1.34 ± 0.001^a^	1.36 ± 0.002^a^	1.24 ± 0.00	1.25 ± 0.00	1.25 ± 0.00
Polyphenols (g L^−1^)	0.41 ± 0.03^a^	0.42 ± 0.03^a^	0.40 ± 0.02^a^	0.64 ± 0.01^a^	0.62 ± 0.01^a^	0.63 ± 0.01^a^
___OD_540_	0.142 ± 0.008^a^	0.163 ± 0.009^b^	0.154 ± 0.006^b^	0.123 ± 0.005^a^	0.129 ± 0.004^a^	0.127 ± 0.007^a^
LAB colonies (log10°CFU mL^−1^)	7.31 ± 0.204^a^	6.84 ± 0.056^b^	6.82 ± 0.058^b^	0	0	0

All data were analyzed by Duncan's multiple range test, different letters in the same row indicate a significant difference was existing in each group (*P *<* *0.05, *n *=* *3).

Compared with various indexes on physical–chemical composition in control group, the turbidity (OD_420_) of the optimal group increased slightly and remained constant in 6 months. The total sugar concentration of the optimal group basically remained the same; in contrast, the total sugar concentration of the control group decreased by 8.9% at the end of the 6th month. The ice wine pH was maintained at 4.0 in the optimal group but pH of the control group slightly increased about 0.3. It was demonstrated that the ice wine had been totally spoiled. With SO_2_ concentration decreasing from 47.73 mg L^−1^ to 30.1 mg L^−1^, LAB activity in the control group intensified and resulted in a considerable increase (40.4%) in volatile acidity. In addition, the changes in alcoholic degree and polyphenol concentration in the two groups were not significant. Besides these, the lysozyme-combined antibacterial system did not lead to ice wine browning since color density in the optimal group was stabilized at 1.25 during storage. Furthermore, the change in ΔOD_540_ in the control group was irregular; comparatively, it was consistent in the optimal group. LAB activities were under control in the optimal group during the 6 months. However with a slight decrease, about 10^7^ CFU mL^−1^ LAB colonies were clearly observed in the control group at the end of 6 months.

## Conclusions

In the beginning, LAB counting method was modified for the practical maneuverability of quadratic rotation-orthogonal composited design. Min-max normalization was carried out to conduct data conversion and to evaluate the synthesis score of the two different indexes, namely, log10 CFU mL^−1^ (LAB inhibition) and ΔOD_540_ (protein stability), meanwhile, a multiple regression model for microbiological experiments was established. Afterward, with the response surface methodology and the ridge analysis, lysozyme-combined antibacterial system, which is special for ice wine, was developed and optimized. The main physical–chemical compounds of optimal groups and control groups were compared during 6 months of aging; the results indicated that lysozyme-combined antibacterial system positively influences physical–chemical compositions and stabilized Italian Riesling ice wine for a long time. In conclusion, as a new technique to reduce SO_2_, lysozyme-combined antibacterial system can be established to stabilize ice wine by adjusting four parameters before aging process. However, further details should be obtained regarding the evolutions of volatile compositions during aging. In addition, the correlated level between different phenolic compounds and lysozyme needs to be investigated as well.

## Conflict of Interest

None declared.
